# Comparative study of composition, antioxidant and antimicrobial activity of two adult edible insects from Tenebrionidae family

**DOI:** 10.1186/s13065-020-00707-0

**Published:** 2020-09-09

**Authors:** Daniel R. Flores, Luz E. Casados, Sandra F. Velasco, Ana C. Ramírez, Gilberto Velázquez

**Affiliations:** 1 Chemistry Department, University of Guadalajara, 1421 Marcelino García Barragán Blvd, 44430 Guadalajara, Jalisco Mexico; 2 Life and Science Division, Food Department, University of Guanajuato, Campus Irapuato-Salamanca, Km 9 carretera Irapuato-Silao ap 311, 36500 Irapuato, Guanajuato Mexico

**Keywords:** *T. molitor*, *U. dermestoides*, Antioxidant activity, Antimicrobial activity, Protein hydrolysates

## Abstract

In the case of Tenebrionidae family insects, studies focus on larval stage, leaving a lack of information regarding other stages. Therefore, this study was performed in order to understand the differences between the nutritional composition and the bioactivity of two species of this family in their adult stage, fed with a specific diet. Adult beetles of both species were defatted, lyophilized and protein extracted with buffer. Proximal and phytochemical analysis of the extracts of each insect were performed, along with protein extract and hydrolysis analysis by Tris-Tricine and Tris Glycine SDS PAGE. This analysis showed that *T. molitor* contained more protein and fat than *U. dermestoides* but contained less crude fiber. The protein extraction was made with PBS, where 130 and 45 kDa bands showed predominant for *U. dermestoides,* and less protein was present for *T. molitor*. Antioxidant and antimicrobial activities of the enzymatic protein hydrolysates and protein crude extracts were determined. Presence of protein associated with the antioxidant activity were found in both insects. Nonetheless *U. dermestoides* had a higher antioxidant activity with the protein extract in contrast with the higher antioxidant activity shown by *U. dermestoides* once the extracts were digested. After proteolysis, protein extracts showed an increasing antioxidant activity, plus, the ability to inhibit microbial growth of *Proteus*, *Shigella* and *Bacillus*. Insect protein hydrolysates with protease open the possibility for the use of these beetles as new sources of encrypted peptides for microbiological control once characterized.

## Introduction

Entomophagy, the consumption of insects, has been promoted as an alternative sustainable protein source for humans and animals [1]. Insect consumption by humans is an old, historically and geographically, widespread phenomenon. The use of edible insects varies greatly by local preference, sociocultural significance, and region [2]. Recently, European countries have an increased interest in studying insects as potential food sources, but it is still a challenge to gain acceptance in society [3, 4]. However, in Latin American cultures, especially in Mexico, the consumption of insects is a tradition rooted in the local cuisine; clear examples of this is the consumption of the grasshoppers, ants, and worms. Despite the early acceptance of entomophagy in Mexico, there are not enough studies which promote the use of different edible insects whose cultivation is simple, practical and under controlled conditions which ensure safe consumption [5]. *Tenebrio molitor* (*T. molitor*) and *Ulomoides dermestoides (U. dermestoides)* beetles, whether in larval or adult stage, are harmless, non-toxic and do not bite. These animals invade barns and are considered a plague by many farmers, but when kept in captivity they can be fed with a great variety of foods such as: wheat flour, maize or oats; barley and wheat bran, remnants of meat, dead insects and decaying trees. Though in terms of hydration, fruit or vegetables are enough to feed them. *T. molitor* and *U. dermestoides* belong to family Tenebrionidae, and are distributed all over the planet, especially in regions with temperate climates. Their sizes varies between 1 and 30 mm. Although insects are known for their diverse nutritional properties, specially associated with their high percentage of protein and/or high content of fatty acids (mono and polyunsaturated), they are also rich in minerals such as: copper, iron, magnesium, manganese, phosphorus, selenium, zinc, vitamins such as: riboflavin, pantothenic acid, biotin and folic acid. Authors like Nowak et al. [32] point out that existing information on the contribution of insects to nutrient intake is still too scarce. While, in some extent family Tenebrionidae has been characterized in terms of nutritional value [6, 7], protein production [8], cytotoxic and genotoxic activity [9, 10], and some nutraceutical properties [11–13]. Aforementioned studies are focused only in the larvae’s stage of the insect. This leaves a deficiency regarding the nutritional information and properties associated with the adult stage of the family Tenebionidae. Even more, there is practically no information comparing insects of the same family when they are fed a specific diet. The present study was conducted to understand the differences between the nutritional composition, and bioactivities of two adult edible insects: *T. molitor* and *U. dermestoides*.

## Materials and methods

### Methods of edible insect preparation

*Ulomoides dermestoides* Chevrolat [13] (= Palembus (Ulomoides) dermestoides, = Martianus dermestoides) it’s an arthropod of the order Coleoptera, family Tenebrionidae, tribe diaperinae, genus Ulomoides, species *U. dermestoides*, originary from the southeast of Asia. The taxonomical identity of *U. dermestoides* was obtained following the keys published by Kim & Jung [14]; *Tenebrio molitor* [15] it’s an arthropod of the order Coleoptera, family Teneobrinidae, species *Tenebrio molitor*, originally from Europe.

The taxonomical identity of *Tenebrio molitor* was obtained following the keys published by the National Science Foundation Grants DRL 0089283, DRL 0628151, DUE 0633095, DRL 0918590, and DUE 1122742. Additional support was granted by the Marisla Foundation, UM College of Literature, Science, and the Arts, Museum of Zoology, and from Information and Technology Services at University of Michigan. Both identifications were later validated by Dr. J. Luis Navarrete Hereida responsible of the scarabaeidae and entomology collection at the reservoir from the Botany and Zoology department of the University of Guadalajara’s Center of Biological and Agricultural Sciences (CUCBA); CZUG collection. CUCBA, SNIB CONABIO database, Project EC017 y CE022. México, D. F.

Specimens from the Tenebrionidae family: Adult beetle *T. molitor* (Linnaeus, Coleoptera: Tenebrionidae) and adult beetle *U. dermestoides* were kindly donated from the identified taxonomic reservoir by Dr. J. Luis Navarrete Hereida and the offspring was deposited in a plastic container where the temperature was maintained between 25 and 30 °C, once at the adult stage, insects were separated into different deposits. Food consisted of a mixture of oats and thin slices of apple (approximately 3.2–4.8 mm thick) every third day as source of hydration [11]. Insects were fasted for approximately 48 h to clear their gastrointestinal tract of any residual food before they were collected. Then they were defatted with hexane for 2 h and set to solvent evaporation. Finally, the samples were frozen with liquid nitrogen and stored at − 70 °C for further experimentation.

### Reagents

2,2-azino-bis (3-ethylbenzothiazoline-6-sulfonic acid) (ABTS) and 2,2-diphenyl-1-picrylhydrazyl, (DPPH); *Aspergillus oryzae* protease was purchased from Sigma-Aldrich. All of the other chemicals used were of analytical grade. Dialysis membranes with 1 kDa and 10 k cut-off were purchased from Spectrum Lab. Culture medium for bacterial growth was Tryptic Soy Broth (TSB) and Tryptic Soy Agar (TSA) from Becton–Dickinson.

### Proximate analysis

All samples were lyophilized and preserved in a freezer at − 80  C (model SC-10 N, brand Scientz). The proximal analysis was performed at the Laboratory of Quantitative Chemical Analysis of the Chemistry Department in the University of Guadalajara´s Center of Exact Sciences and Engineering (U de G). Following the methods by the Association of Official Analytical Chemist [16], such as AOAC-934.01 for moisture, AOAC-928.08 for Nitrogen content and Protein content estimation, AOAC-920.39 for Fat/Crude fat, AOAC-962.09 for crude fiber and AOAC-923.03 for Ash content.

### Qualitative phytochemical analysis

Extracts of each insect (0.1 g/ml) were subjected to preliminary phytochemical screening (acidic, alkaline and neutral aqueous systems were used when necessary) by standard methods [17] for detection of the following constituents: tannins [18], alkaloids, triterpenoids, carbohydrates, steroids [19], saponins, glucosides [20], flavonoids [21], and anthraquinones [22].

### Protein extract preparation

Proteins were extracted as follows: 1.0 g of insects (previously treated as above mentioned) were weighted and extracted with buffer Phosphate-Buffered Saline (PBS) (for most cases unless specified) in 1:9 ratio (insect: buffer), then each sample was sonicated for 5 min (during a 30 s interval on, and, 15 s off). The sonicated extract was centrifuged at room temperature for 10 min at 8000*g*, and supernatant was collected; when necessary, acetone (4:1 acetone: supernatant) or ammonium sulfate (30% saturation) was used for the concentration of protein extract, followed by resuspension in PBS. All the supernatants (either concentrated or not) were subjected to overnight dialysis (using a 10 kDa cut-off membrane). Supernatants were stored at 4 °C to further analysis.

### Tris glycine and Tris tricine SDS-PAGE protein electrophoresis

Analysis of the extracts were performed on 12% of SDS-PAGE electrophoresis gel according to the protocol established by Laemmli (Tris Glycine system). A Mini-Protean BioRad (BioRad, Hercules, CA, USA) electrophoresis system with 20 mA was used. Samples were mixed with Laemmli buffer (1:1, v/v) and heated at 90  C for 5 min prior to the electrophoresis. Gels were stained with Coomassie Blue R-250 using the thermo molecular marker (page ruler unstained broad range protein ladder and page ruler unstained low range protein ladder). When necessary (peptide visualization) a Tris Tricine system on 16% of SDS-PAGE was performed according to the protocol of Schägger H [23]. Protein quantification was done using Bradford method [24].

### Protease digestion

Samples were treated with *Aspergillus oryzae* (*A. Oryzae*) protease from sigma (1:200) (protease: sample) in a 500 μL reaction volume. Untreated samples, buffer alone, or enzyme solutions were used as controls. Treated samples and controls were incubated at 37 °C for 24 h. They were then dialyzed on a 1 kDa cut-off membrane (to avoid loss of peptides) against PBS for 2 h at 4 °C, before its use on antioxidant and antimicrobial activity assays.

### ABTS radical scavenging activity

ABTS Assay (ABTS^+^) was determined according to Re et al. [25] with some modifications. The radical ABTS^+^ was chemically generated using ammonium persulfate, diluted in deionized water until a final concentration of 7 mM. The solution was diluted to reach an absorbance around 0.7 at 750 nm. In a 96-well microplate, 20 µL of sample and 280 µL of ABTS^+^ solution was mixed. The absorbance change (750 nm) was measured after 15 min of incubation at room temperature. The percentage scavenging activity was calculated according to Eq. 1:

$$Scavenging activity \left( \% \right) = \left( {\frac{{A_{control} - A_{sample} }}{{A_{control} }}} \right) \times 100$$

where: A_sample_ is the absorbance of the ABTS^+^ solution with the sample; and A_control_ is the absorbance of the ABTS^+^ solution without the sample.

### Antimicrobial activity

Samples were assayed by the well diffusion method according to de la Fuente Salcido et al. [26, 27] with some modifications. Briefly: twenty-five mililiters of freshly prepared sterile Tryptic Soy Agar (cooled to 40–50 °C) was poured into sterile Petri dishes containing 115 µL (1 × 10^9^ cells/mL) of the following bacteria: *Micrococcus luteus*, *Bacillus aerius*, *Samonella* spp., *Bacillus wiedmannii*, *Streptococcus canis*, *Enterococcus casseliflavus*, *Bacillus cereus* strain JCM 2152, *Bacillus cereus* strain 183, *Listeria monocytogenes* and *Listeria innocua* and *Vibrio parahaemolyticus*. These bacteria are maintained in a stock collection of microorganisms in our laboratory. Once the mixture solidified, wells of 6 mm in both diameter and depth were made on the plates using sterile cork borers. 90 µL of the prepared sample was then dispensed into the wells, allowed to equilibrate at room temperature for 30 min, and then incubated overnight at 37 °C. Zones of growth inhibition (in mm), were measured as the diameter of the clear zones around each well, and, then compared with the antimicrobial standards. All the experiments were done in triplicates and the data is presented as mean values.

### Statistical analysis

The experiments shown are the mean value ± standard error (SE) of the data from at least three experiments. Statistical analyses were performed using Graph Pad Prism 8 statistical software. Differences with p values of less than 0.05 were considered significant.

## Results and discussion

### Proximate analysis

Insects are well known to be rich in nutrients: proteins, lipids, carbohydrates, vitamins and minerals [28, 29]. There are several reports suggesting that insects could be an excellent source of protein, since their availability in nature is very abundant and their breeding economically feasible [30]. Composition of adult beetles of *T. molitor* and *U. dermestoides* are shown in Table 1. The highest content of protein and fat was found in *T. molitor*, while in terms of crude fiber *U. dermestoides* had the highest value. There is no statistical difference between adult beetles in term of ash contents. It is important to notice that all results were expressed in dry mass in order to have a comparative value for other studies.

**Table 1:** Physicochemical composition of Tenebroideae beetles *T. molitor* and *U.dermestoides*

	*U. dermestoides*	*T. molitor*
Ash	2.12 ± 0.71	3.24 ± 1.48
Protein (N × 6.25)	40.36 ± 0.79 *	54.86 ± 1.70 **
Fat	8.33 ± 0.69 *	12.50 ± 1.46 **
Crude fiber	49.17 ± 0.32 *	26.63 ± 0.69 **
NFE	0.02 ± 1.38	2.77 ± 0.94

Values are expressed as mean ± standard deviation (n = 3). All data are expressed as g/100 g dry mass, N: Nitrogen

*At the exponential, in the same row, show there are significant differences (p-value < 0.05)

The protein content found in this work for *T. molitor* was 54.86 ± 1.70% which fits within the range reported in previous studies [31–33]; while the protein found for *U. dermestoides* in this work is slightly lower than the 48.31% reported by Zhou and Chen [34]. In contrast with the protein content found in other insects’ orders, it fits within the range of grasshoppers/locust 40–74.28%, Lepidoptera (wax moth and butterflies) 14 –68%, Hemiptera 42–74%, Orthoptera (crickets) 55.65–75% and Diptera (common housefly) 55–70% [35–38]. Our data allows us to support what is suggested in previous studies such as the ones published by Payne CLR et al. and Halloran et al. [29, 30]. Which expose the viability for the use of insects to transform low value organic by-products into high value foods with very low greenhouse gas emissions.

In terms of fiber content, *U. dermestoides* presented a very high value (49.17 ± 0.32%) compared with the 22.1% reported by Jasso-Villagomez et al. [39]. When compared with other insect orders like Orthoptera, the difference in fiber content for *U. dermestoides* is more significant, for example, the grasshopper *C. rosea* has 12.38% of fiber content, while bush criket *B. orientalis* has only 8.75% [33, 38].

### Qualitative phytochemical analysis

In the qualitative phytochemical analysis (Table 2), the presence of some secondary metabolites commonly associated with the antioxidant activity (tannins or flavonoids) were not found, neither in *T. molitor* nor *U. dermestoides*, this analysis was performed after the defatting of the insects in order to confirm that the antioxidant activity evaluated was only due to the protein fraction. Carbohydrates and reducing sugars were detected in both insects. Glucosides, flavonoids, alkaloids and steroids were absent using the procedures indicated for each test. Proteins were detected using Bradford reagent with both beetles; there was a major color intensity on the qualitative test of *T. molitor* in comparison with the test of *U. dermestoides*, in agreement with the nitrogen content estimated by the proximal analysis. The stable foam formation suggested the presence of saponins. No qualitative tests were considered as positive in this work for alkaloids, steroid or anthraquinones [40].

**Table 2:** Qualitative phytochemical results of Tenebroideae beetles *T.molitor* and *U. dermestoides*

Phytochemical constituents	Test performed	Insect aqueous extract	
		*U. dermestoides*	*T. molitor*
Tannins		−	−
Saponins	Superficial foam	++	+
Glucosides	Keller-Kiliani	−	−
	Borntrager’s	−	−
Carbohydrates	Molisch’s	+	+
	Benedict’s	+	+
	Fehling’s	+	+
Flavonoids	Lead acetate	−	−
	Salkowski’s:	−	−
	Shinoda	−	−
Proteins	Bradford	+	++
Alkaloids	Dragendorff’s:	−	−
	Mayer’s	−	−
	Wagner’s	−	−
	Hager’s:	−	−
Steroids	Liebermann Burchard’s	−	−
Anthraquinones		−	−

Values are expressed as positive (+), very clear result (++), negative (−); not applicable (NA). Acidic extraction and alkaline extraction were used for alkaloids and steroids (nevertheless, no positive results were detected)

### Protein extract preparation

**Fig. 1:**
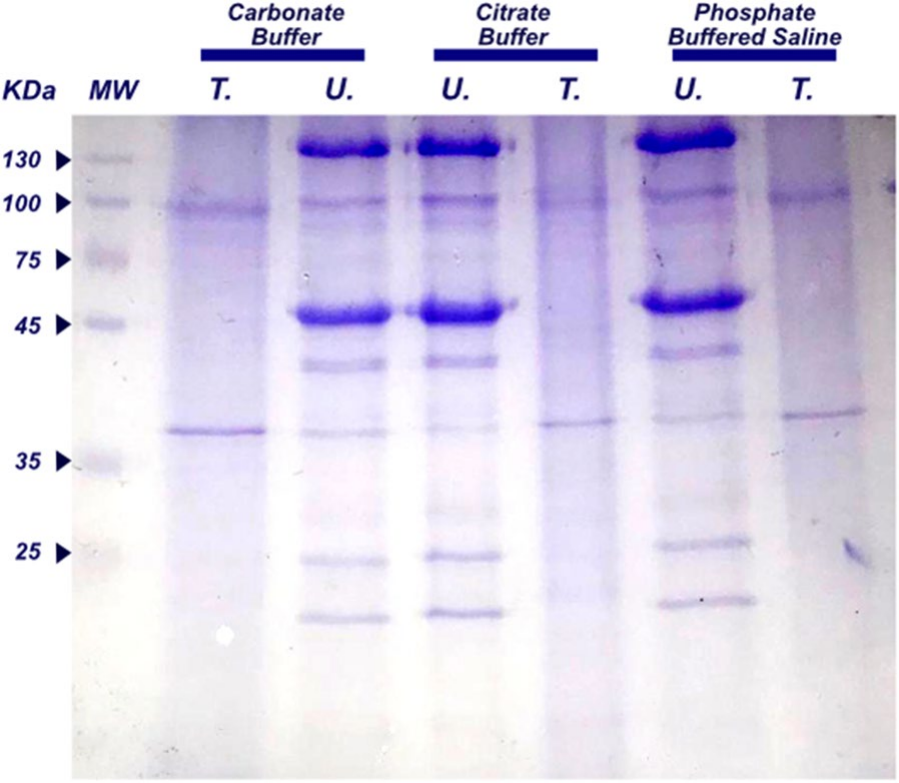
SDS-PAGE of protein extracts from *T. molitor* and *U. dermestoides adult* insects. A 15% of Tris Tricine SDS-PAGE gel is presented. There was no significant difference among the buffers tested in terms of protein band detection. Elecrophoresis shows better resolution of the bands for *U. dermestoides* extract in comparison with *T. molitor’s*. Protein extracts show that the number of peptides present in the direct extraction is practically absent. MW: Protein Molecular Weight Marker in KDa

Three buffering systems were used to evaluate the best extraction conditions for *U. dermestoides* and *T. molitor* insect proteins. Despite the differences in pH of the extraction systems (Citrate buffer pH 5, carbonate buffer pH 9 and PBS buffer pH 7), polyacrylamide gel denaturing electrophoresis (SDS PAGE), showed no significant differences in band patterns for the extracts (Fig. 1). We then decided to use PBS as a buffer for protein extraction. Spite the several replicates obtained by the sonication method (regardless of the buffer) on the extraction of *T. molitor*, the electrophoretic profile always showed few bands.The protein profile revealed by electrophoresis of both insects’ crude extracts showed very different patterns and molecular weight bands. For *U. dermestoides* most of the bands center around 45 kDa; but there is a presence of low molecular bands between 25 and 15 kDa, and a very strong band around 130 kDa. By the other hand, very few bands were distinguishable in the *T. molitor* protein crude extracts, two strong bands: one near to 95 kDa and the other around 37 kDa. This can be explained by the conditions used for the extraction in this work, where the selected buffers did not count with the presence of caothrophic agents such as urea or detergents as SDS or Triton, which contributed to the low protein extraction of *T. Molitor*.

These parameters where employed in urge to minimize further effects on the protein quantifications assays such as the digestion of crude extracts. It’s worth mentioning that previous works reporting any electrophoretic profile where focused on the larvae stage of the insect [37] rather the adult stage, and, applying rougher extraction conditions (prolongated agitation [42], or extended sonication time [43]), conditions that we avoided in order to restrict the protein degradation in this work. Studies such as Zielińska et al. [11], state that the electrophoretic protein profile for a specified extraction may vary greatly due to changes on the conditions employed.

Although the methodology for protein extraction was not the same as in the protein profile previously reported for *T. molitor*, we were able to detect some of the bands present on those studies [12, 34, 35]; for example, the bands in the range of 32–95 KDa in *T. molitor* were also obtained in this work, except no bands above 95 KDa were detected as aforementioned studies report. [41].

### Protease digestion of PBS extract

**Fig. 2:**
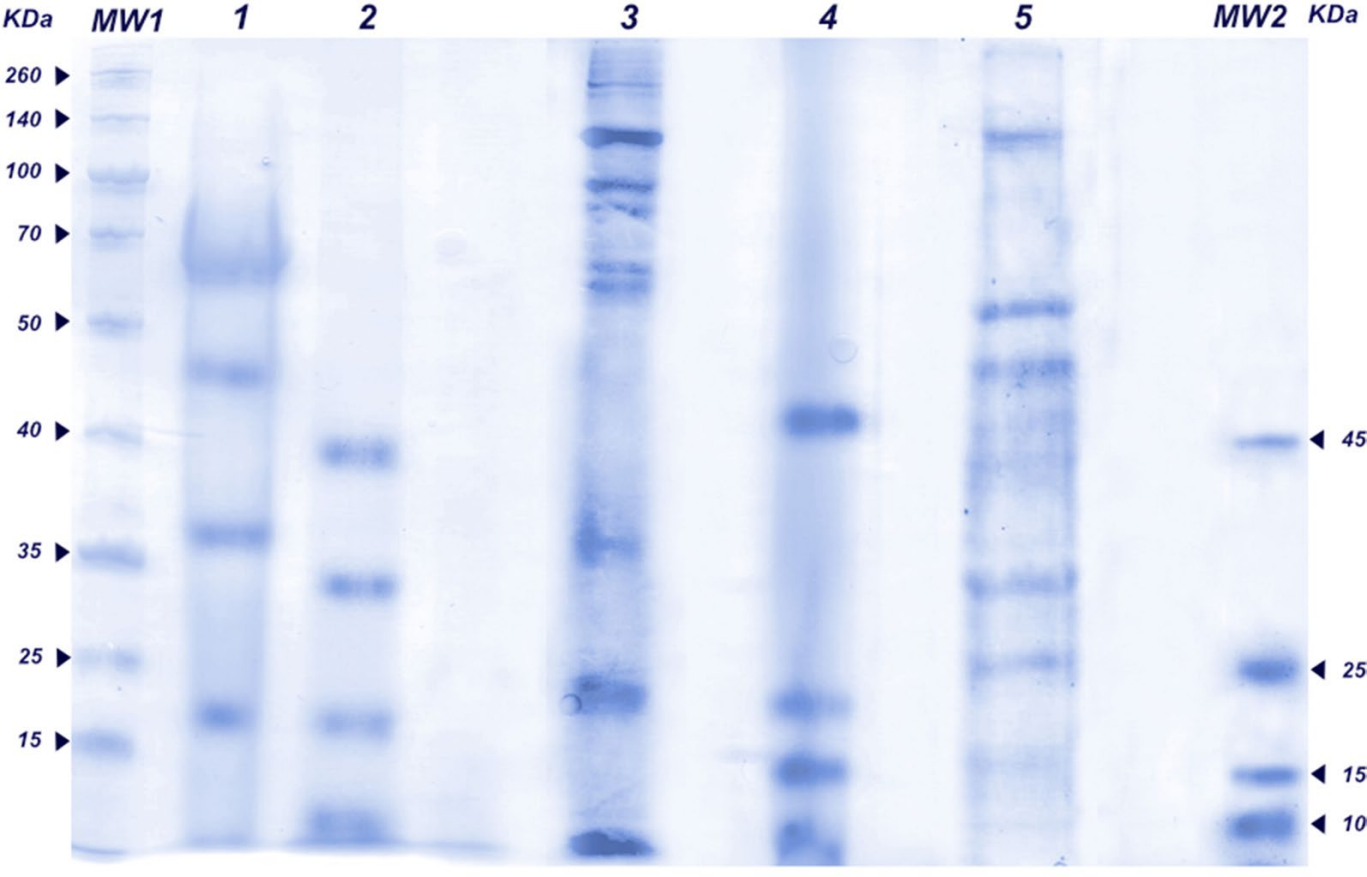
Tris Tricine SDS-Gel with the hydrolysate *U. dermestoides* and *T. molitor* extract with *A. oryzae* protease. MW1. Protein marker (numbers indicate KDa). Lane 1. Control of autoproteolysis with *A. oryzae* protease under the same conditions of digested extracts. Lane 2. Digested extract of *U. dermestodies* with *A. oryzae* protease. Lane 3. Non-digested extract of *U. dermestoides* in PBS. Lane 4. Digested extract of *T. molitor* with *A. oryzae* protease. Lane 5. Non-digested extract of *T. molitor* with PBS. MW2. Peptide protein marker (numbers indicate KDa)

In order to explore the diversity of encrypted peptides we decided to achieve proteolysis of the PBS extracts from the *U. dermestoides* and *T. molitor* insects. Using an *A. oryzae* protease containing both endoprotease’s and exopeptidase’s activity, the PBS extracts were digested, and the peptide profile was analyzed with an optimized gel (Tris-Tricine SDS-Gel) for peptides [20]. Figure 2 shows in lane 1 the autoproteolysis control and in lane 2 the digested extract of *U. dermestoides*. It is possible to observe that the digested extract presents two major bands of peptides between 15 and 35 kDa with differential bands compared to the autoproteolysis control, there is also a digested fraction under 15 kDa present in the gel. Meanwhile *T. molitor* digested extract showed a range of 1.5–25 kDa peptide profile. No bands above 40 KDa were detected for any of the digested extracts, and the protein profile is completely different respect to the total protein extracts.

Due to the use of a protease with both endo- and exo- peptidase activities, as it is A. Oryzae, it is likely that the liberated fragments are attributable to an exhaustive proteolysis of the hemolymph proteins of the insects. It is reported for T. Molitor a defensin (small cationic cysteine rich peptide) [42], with a molecular weight below 10 kDa, known as tenecin-1 [43]. More experimental data is required for a detailed characterization of the peptides liberated throughout the process applied by fungal proteases as A. Oryzae, however, the approach of the encrypted peptides obtention process by this kind of proteases looks promising [42, 44].

### Total antioxidant capacity of protein insect extracts

**Fig. 3:**
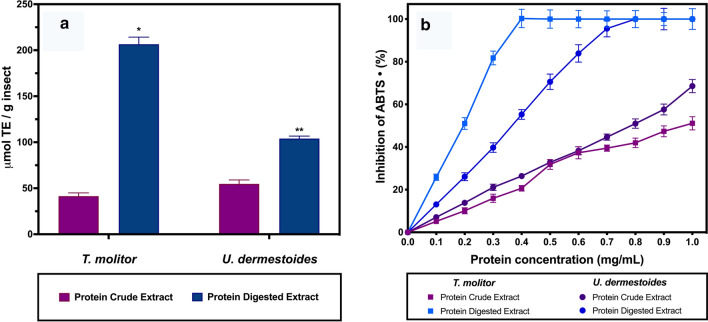
Total antioxidant activity of the protein extract from *U. dermestoides* and *T. molitor.* **a** Comparison of the antioxidant activity of protein extracts before and after digestion with protease. The asterisks show that there is a statistical difference between the protein digested extract at 95% of confidence. **b** Inhibition of ABTS radical assay with the non-digested and digested protein extract

The antioxidant activities of the extracts were expressed as micromoles of Trolox/g insect (Fig. 3a). Protein concentrations were adjusted to obtain similar amounts of protein for the antioxidant activity test. If we compare PBS extract from *U. dermestoides* and *T. molitor* there was no statistically significant difference at 95% confidence interval by a student’s *t* test. Although, the antioxidant activity found for *Tenebrio* protein extract (41.4 ± 2.3 µmol Trolox/g) and *U. dermestoides* protein extract (54.8 ± 3.1 µmol Trolox/g) was less than the reported for fruits such as strawberry (358 µmol Trolox/g), blueberries (348.25 µmol Trolox/g) and pomegranate peel and pulp (4.6–6.3 mM Trolox/g); higher than the water-soluble extracts of grasshoppers (2.55 + 0.05 µmol Trolox/g), silkworm (2.48 + 0.19 µmol Trolox/g), crickets (2.37 + 0.03 µmol Trolox/g) and fresh orange juice (0.40 ± 0.01); and similar to the activity found in freeze-dried peel of purple star apple (3310.95 μM Trolox/100 g DW) and yellow cashew (3322.31 μM Trolox/100 g DW) [43–45]. Nevertheless, the activity we obtained with the extract is referred exclusively to protein fraction per insect gram. Moreover, when a digestion with *A. oryzae* protease was performed on the protein crude extract, there was a significant increase on the total antioxidant capacity for the sample, reaching 206 ± 8.5 µmol Trolox/g for *T. molitor* extract and 104 ± 6.2 µmol Trolox/g for *U. dermestoides* extract respectively.

We evaluated the ABTS radical inhibition percentage (Fig. 3) using the insect protein extracts (PBS and protease digested). The results showed that a 100% of the ABTS radical inhibition was reached at approximately 0.4 mg/mL of protein for the digested extract of *T. molitor* and it takes 0.8 mg/mL of protein for the digested extract of *U. dermestoides*. When the PBS protein extracts were used no complete radical inhibition for either insect was achieved, under the kinetic conditions used. Even though both insects belong to the same family and were fed under the same diet, the kinetic profiles of the antioxidant activity were very different. In the case of the *U. dermestoides* digested extract, it took almost twice the amount of protein in order to achieve a 100% of the ABTS radical inhibition.

### Protease digestion of PBS extract for antimicrobial activity evaluation

We decided to test if the hydrolysates obtained with the *A. oryzae* protease were able to inhibit the growth of some of the bacteria on a well diffusion method test. Only three microorganisms among the tested in our assay, presented inhibition by the digested extracts. Two gram negative microorganisms (*P. vulgaris* and *Shigella flexnerii*) and one gram positive (*Bacillus spp*) presented inhibition by the digested protein extracts of both insects. Results are summarized in Table 3. Extracts digested with protease open the possibility for the use of these beetles as a new source of encrypted peptides for microbiological control once characterized.

**Table 3:** Antimicrobial activity of the enzymatic hydrolysates of protein extract from *U. dermestoides* and *T. molitor* against selected microorganisms

Protein digested extract	Diameter of growth inhibition zone (mm)				
	*P.* *vulgaris*	*S. flexnerii*	*Bacillus* *t. subsp.* *Tenebrionis*	*Bacillus* *t. subsp.* *kenyae*	Enzymatic control
*U. dermestoides*	9 (+++)	11 (++++)	13.5 (++++)	7.7 (+++)	< 1 (−)
*T. molitor*	7.5 (+++)	12 (++++)	8.5 (+++)	5.5 (++)	< 1 (−)

Each value is expressed as mean (n = 3) and standard deviations were less than 5%. No antimicrobial activity (−), inhibition zone < 1 mm. Slight antimicrobial activity (+), inhibition zone 2–3 mm. Moderate antimicrobial activity (++), inhibition zone 4–5 mm. High antimicrobial activity (+++), inhibition zone 6–9 mm. Strong antimicrobial activity (++++), inhibition zone > 9 mm. Standard deviation ± 0.4 mm. The protein crude extracts showed no inhibition zone

## Conclusion

High fiber and protein content values were obtained with the diet supplied to insects during this work. Antioxidant activity for the protein crude extracts of *T. molitor* and *U. dermestoides* showed values around 41.4 and 54.8 µmol Troxol/g respectively, however once the extracts were digested with *A. oryzae* protease, the antioxidant activities increased to 206 for *T. molitor* and 104 for *U. dermestoides.* The digested extracts presented different kinetic patterns on the inhibition of ABTS radicals, where *U. dermestoides* needed twice as digested protein extract compared to *T. molitor*. These also showed antimicrobial activity against *P. vulgaris*, *S. flexnerii* and *Bacillus spp.* All this data suggests that *T. molitor* and *U. dermestoides* are an excellent possibility as sources of fiber and protein moreover their antioxidant properties make them suitable as nutraceutical food.
